# Understanding the Potential of Light Absorption in Dots-in-Host Semiconductors

**DOI:** 10.1021/acsphotonics.4c00760

**Published:** 2024-07-24

**Authors:** Miguel Alexandre, Hugo Águas, Elvira Fortunato, Rodrigo Martins, Manuel J. Mendes

**Affiliations:** Department of Materials Science, NOVA School of Science and Technology and CEMOP/UNINOVA, i3N/CENIMAT, Campus de Caparica, 2829-516 Caparica, Portugal

**Keywords:** quantum dots, nanostructured semiconductors, multi-band solar cells, interband absorption, k.p method

## Abstract

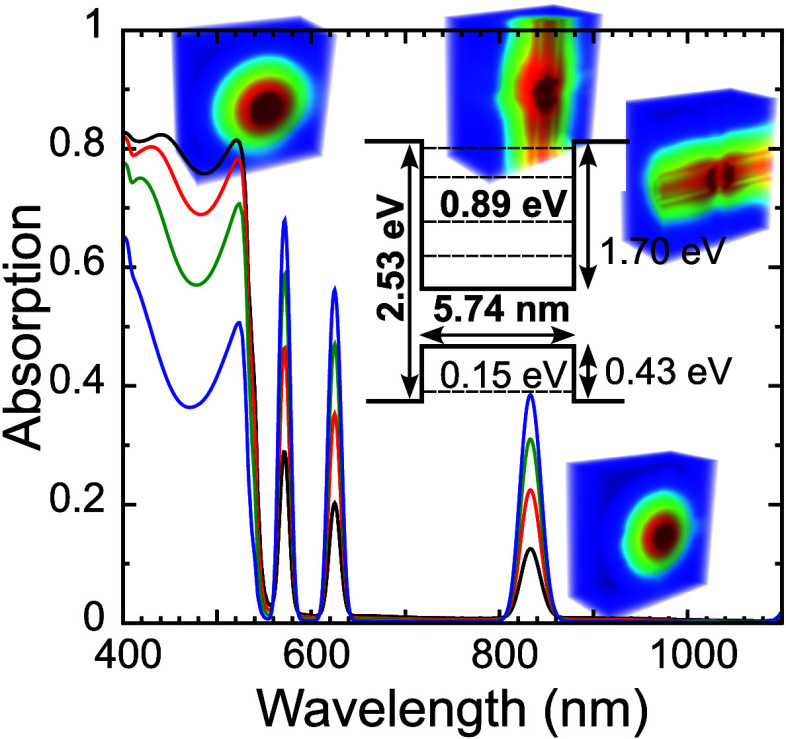

The outstanding physical properties of dots-in-host (QD@Host) hetero semiconductors demand detailed methods to fundamentally understand the best routes to optimize their potentialities for different applications. In this work, a 4-band k.p-based method was developed for rock-salt quantum dots (QDs) that describes the complete optical properties of arbitrary QD@Host systems, trailblazing the way for the full optoelectronic analysis of quantum-structured solar cells. Starting with the determination of the QD bandgap and validation against well-established literature results, the electron transition rate is then computed and analyzed against the main system parameters. This is followed by a multiparameter optimization, considering intermediate band solar cells as a promising application, where the best QD configuration was determined, together with the corresponding QD@Host absorption spectrum, in view of attaining the theoretical maximum efficiency (∼50%) of this photovoltaic technology. The results show the creation of pronounced sub-bandgap absorption due to the electronic transitions from/to the quantum-confined states, which enables a much broader exploitation of the sunlight spectrum.

## 1 Introduction

The excellent optoelectronic properties of Colloidal Quantum Dots (CQDs), arising from their small size (∼few nanometers), present a nexus for research in different areas, such as light emitting diodes (LEDs), luminescent up/down-shifting, quantum computing, microscopy, and biology.^1−7^ At such small sizes, quantum effects start to dominate, and thus the material enters a strong electronic confinement regime where energy levels become discrete and highly narrow transitions become possible.^2,8^ These effects are highly dependent on the QD material, size, and surrounding medium; thus demanding the careful control of all of these properties.^1,9^

One particular research area that has shown increasing interest in the outstanding properties of CQDs is solar cells.^3,10−15^ By incorporating CQD arrays into the structure of a photovoltaic absorber material (QD@Host), essentially creating a superlattice, it is possible to produce significant below-bandgap absorption (and consequently photocurrent) without voltage loss,^11,12,16^ thence improving photovoltaic (PV) efficiency beyond the well-known Shockley-Queisser limit. Furthermore, it was recently revealed that, despite the strong confinement provided by such nanostructures, their absorption coefficient can reach values comparable to^17,18^ (or even surpassing^19^) the absorption of conventional bulk semiconductors.

In particular, PbS CQDs have been shown to have excellent structural compatibility with perovskite host materials,^3,20,21^ with the latter also showing amazing optoelectronic properties that granted perovskite solar cells incredible efficiencies reaching 25.6%,^22−25^ thus underlining a path worth exploring for QD@Host applications. Furthermore, combining these QD@Host absorbers with advanced light-trapping (LT) techniques^26−30^ could pave the way for highly efficient yet flexible PV technology, an important market driver.^31−33^

Detailed models of the optical behavior of QD@Host semiconductors are thus fundamental to accompany the technological developments. Currently, the main simulation techniques are based in quantum-mechanical atomistic simulation methods which are precise, yet quite complex as is the case of density functional theory models^19,34^ that create time and computational constraints while being limited to study highly specific QD systems. Also, infinite potential based k.p envelope function formalisms^34−36^ can provide correct measurements for transition energies upon application of a correction factor to the QD diameter to better model physical boundaries;^34^ however, they do not consider changes from the originally imposed infinite potential barrier. This is problematic for the realistic modeling of practical scenarios, since the QD surface effects are poorly described. For instance, it has been shown that ligand exchange in CQDs can significantly impact the final optical properties,^37^ which is of particular relevance for several QD@Host applications, where the potential difference between host and QD could have a significant impact on the final absorption properties.

In this work, we developed a 4-band envelope function model, based on an empirical k.p Hamiltonian for rock-salt materials (such as PbS, PbSe, and PbTe), which considers the host bandgap, to determine the optical behavior of QDs@Host materials. Thence, it is possible to calculate the absorption coefficient and, via an effective medium approach, the complex refractive index of QD@Host materials with arbitrary QD density and composition. Equipped with this, we performed a complete optimization of the system, considering the application in intermediate band (IB) solar cells which foresees a theoretical maximum efficiency of ∼50% for single-junction devices with the ideal energy bands configuration.^11^ From these optimized results, a single slab of material was studied via a Scattering Matrix Method to determine the overall absorption spectrum. The results revealed that, when embedded into a host material, the QDs absorption gains at photon energies below the host bandgap can be quite pronounced and thereby create a conducive route for highly efficient solar cell applications. In view of that, this work aims at an initial development and analysis from the quantum to the optical domain that can be easily expanded to other levels of modeling, such as the incorporation of electrical device simulations and/or light-trapping schemes.^26,38^

## 2 Results

### 2.1 Energy Level Design and Experimental Comparison

**Figure 1:**
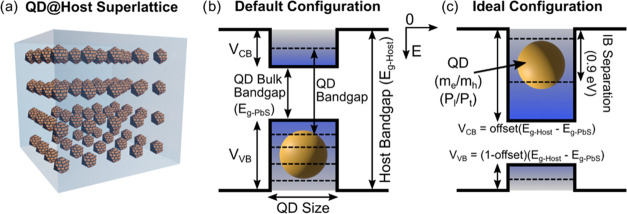
(a) Schematic representation of a QD@Host superlattice system; (b), (c) schematic representations of the band diagram for the 4-band system (each CB and VB is doubly degenerate), with the relevant parameters that are indicated for the default case in Table 1; (b) representation for the default configuration as determined from literature values for the different properties; (c) ideal band configuration, comprising a 2.3 eV host bandgap in which the intermediate band (IB, composed by the energy-aligned QD ground states) is formed at 0.9 eV below the host CB minimum, which maximizes the performance of IB solar cells.^39^

To provide a source of comparison and deeper analysis for the results, we chose to follow a currently well-studied literature example of QD@Host, namely PbS QDs in a wide-bandgap perovskite host, which was shown to have great interfacial properties.^21^Figure 1a shows a schematic representation of the QD@Host superlattice system, while panels (b) and (c) show the 4-band configurations for default and ideal systems, respectively, together with the different relevant parameters necessary for modeling. These parameters can be defined from the constituent materials (summarized in Table 1 termed here the default case). These will be the parameters used unless otherwise stated.

**Table 1:** Default Values for the Different Simulation Properties, Extracted from the References Indicated in the First Line^a^

*m*_e-PbS_ (*m*_0_)^41,42^	*m*_h-PbS_ (*m*_0_)^41,42^	Pt (kg·m/s)^36^	Pl (kg·m/s)^36^	*E*_g-Host_ (eV)^39^	*E*_g-PbS_ (eV)^42^	*V*_CB_ (eV)^43^	*V*_VB_ (eV)^43^
0.08	0.08	4.7e-25	3.4e-25	2.3	0.4	0.4	1.5

a Unless otherwise stated, these are the values used during modeling. Here, *m*_e-PbS_ and *m*_h-PbS_ are the electron and hole effective masses for PbS, respectively. *P*_t_ and *P*_l_ are the transverse and longitudinal components of the momentum matrix elements. *E*_g-Host_ and *E*_g-PbS_ are the bulk bandgaps of the host (here, perovskite) and QD material (here, PbS), respectively, and *V*_CB_ and *V*_VB_ are the size of the potential barrier for the conduction and valence bands, respectively (described in Figure 1).

Furthermore, we also followed the convention of properties that provide the ideal performance for an intermediate band (IB) solar cell.^39^ First, this was used to point out the ideal host material bandgap of 2.3 eV, which then allowed to choose a perovskite configuration and thus determine some of the remaining properties (conduction band potential barrier size, *V*_CB_, and valence band potential size, *V*_VB_, shown in Figure 1b,c as summarized in Table 1. Second, the best-performing band configuration of the IB cell concept places the IB at 0.9 eV below the host CB minimum,^39^ which indicates the optimal placement for the QD ground states pursued in this work (Figure 1c). Quite interestingly, both default and optimal configurations show different band asymmetries (the default has a deeper VB, while the ideal has a deeper CB). This is mostly a consequence of the band alignment provided by the literature values for the perovskite material. In terms of the model, whether there is a deeper CB or VB is mostly irrelevant as the calculations are symmetric for both cases. Nevertheless, this is also a minor concern in practice, as perovskite compounds are well-known to be highly adaptable by playing with their composition,^40^ such that the asymmetry can be reversed by material engineering.

As a first corroboration step in this analysis, we compare the results for the QD bandgap (lowest energy transition, indicated in Figure 1b) with the empirical results by Moreels et al.^44^ This establishes a baseline validity for the model, particularly for comparison of the exciton binding energy.

**Figure 2:**
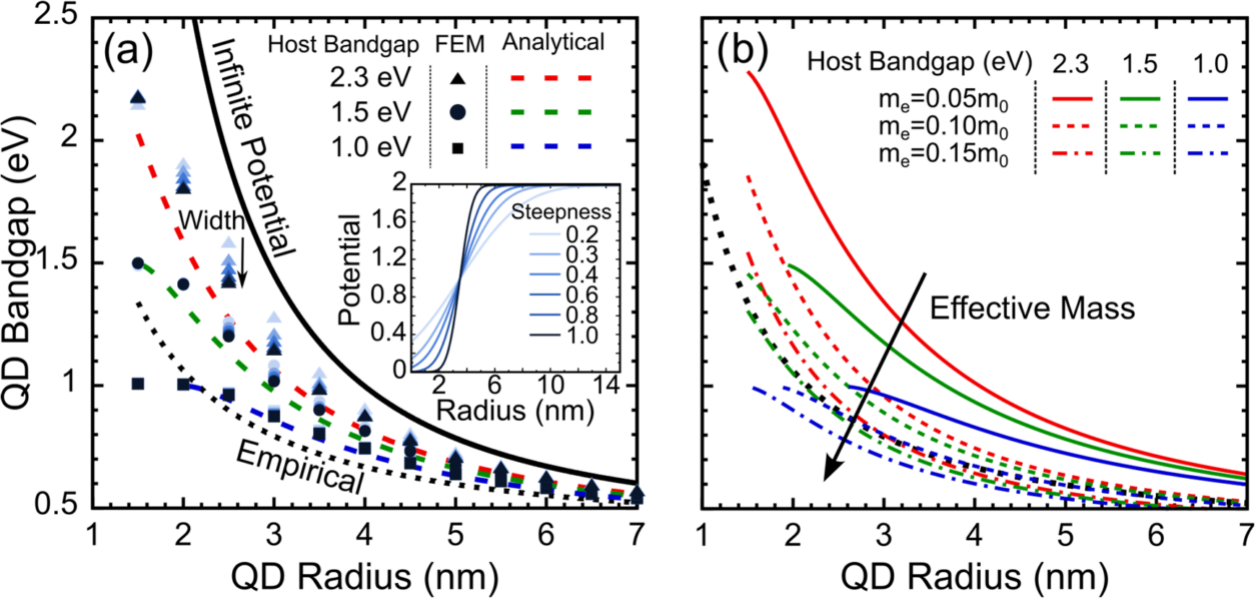
Dependence of the QD bandgap (lowest energy transition) on the QD radius (a) for different host bandgaps (*E*_g_ = 2.3, 1.5, and 1.0 eV dashed red, green, and blue lines, respectively) and an infinite potential (full black line), compared to empirical results (dotted black line) for CQD dispersions. The inset shows the error function potential taken for the dots boundaries for different potential steepness, simulated with the finite elements method (FEM). (b) QD bandgaps for different host bandgap and QD effective mass (*m*_e_ = *m*_h_).

Figure 2 summarizes the global dependence of the QD bandgap on several model parameters, also showing the upper limit (infinite potential) and the empirical results from aqueous CQD dispersions measured by Moreels et al.^44^ There are two main regions to consider. First, for the bigger QD region (radius >4 nm), the bandgap simply tends to the standard bulk value of the QD material (*E*_g-PbS_ = 0.4 eV), and there is a limited dependency on the QD@Host parameters. In the smaller size region (radius <4 nm), the QD bandgap changes more significantly due to the stronger confinement.

Figure 2a shows two sets of simulations. First, the standard analytical solution for different host bandgaps (*E*_g-Host_). Second, a finite element method (FEM) solution implemented using the Deal.II FEM package^45^ (details in Supporting Information Section S2) employing an error function to define the barrier potential, with different degrees of steepness (as shown in the inset in Figure 2a). The infinite potential solution is also shown as the upper limit of the barrier height. This effect is better shown in Figure S2 of Supporting Information Section S3, where it is evident that as the host bandgap increases (also increasing the potential barrier in each band), so does the QD bandgap. Notably, this follows recent results in the literature showing a decrease in the exciton energy when the surrounding material is changed from the Oleic acid (OA) dispersing medium to a perovskite-based compound.^46−50^ Furthermore, as the QD radius decreases, this impact also increases due to the stronger confinement. This decrease, however, is always limited by the host bandgap imposed for the simulations (e.g., a host bandgap of 1 eV limits the first transition energy to 1 eV as seen in Figure 2a) for smaller QD radii.

Considering the empirical result (dotted black line), that should be emulated by higher host bandgap values (i.e., higher barrier height). There is a relevant difference between the simulation and empirical results if a 0.08*m*_0_ effective mass is considered for the PbS. This difference is, however, diminished for an effective mass of 0.15*m*_0_ as shown in Figure 2b. Often here, there are several techniques to mitigate the disparity, such as applying a correction factor to the QD size, thence providing a better model for the physical boundaries^34^ and changes to the band parabolicities.^51^ In light of this, we also made numerical (FEM) calculations of the QD bandgap based on a smoothly changing (error-function-like) potential barrier profile, instead of step-like, as a function of the QD radius (simulation details and convergence tests are described in Supporting Information Section S2). Here, the function steepness (plotted in inset profile of Figure 2a) is determined by the multiplying argument of the error function, a parameter related to the width of the integration of the Gaussian curve that defines how the potential changes in space. This setup is explained in Section S2 of Supporting Information. Lower steepness values result in smoother spatial variation of the barrier walls, and as the steepness increases, the function tends to the step-barrier profile. For lower steepness slower variation of the potential in space, the QD bandgap does increase. At first glance this may seem a counterintuitive behavior; however, upon closer look, this is a simple consequence of a tighter potential exerted in the lower QD levels (easily seen in the inset plot in Figure 2a). This is further emphasized as this difference grows with the increase of the host bandgap.

Figure 2b complements the previous results by adding the effective mass dependency of the QD bandgap. It has been shown that it is possible to improve the quality of the model by defining an energy dependent effective mass.^52^ This is particularly relevant, as the QD itself also exhibits a structural dependency upon size changes,^34^ thus also impacting the structural parameters, such as the effective mass. As can be seen from Figure 2b, for lower QD radii, the effective mass can have a significant effect on the overall bandgap profile. For instance, by changing the effective mass from 0.05*m*_0_ to 0.15*m*_0_ in the 2.3 eV host bandgap system, the bandgap profile of the QD can get significantly close to the empirical results. Furthermore, a higher host bandgap does lead to a more pronounced change in the QD bandgap profile when changing effective mass.

Considering previous experimental results of QD@Host materials, it should be noted that the fabrication of PbS@Perovskite films has been attempted by previous contributions,^46,48,53^ but several challenges still need to be overcome. Namely, the resulting compositions of the materials (highly process-sensitive) have been far from the desired monodispersed arrays of embedded dots, presenting a high level of QDs agglomeration within the perovskite medium, among other issues. Consequently, often the absorption spectra do not have well-defined QD absorption peaks, but one can still provide some comparisons with the results presented here. For instance, in the work of Masi et al.,^47^ the PbS@Perovskite absorption spectra for the indicated 4.7 nm QD diameter gives the first transition peak at 1120 nm (measured). Using that diameter in our model with the referenced properties of the materials (1.55 eV perovskite bandgap, 0.4 eV PbS bandgap, and symmetric potential offsets) results in the first transition peak at 1045 nm, which is close (within 6.7% deviation) to the experimentally observed spectral position. Other recent works of Menda et al.^46^ and Ribeiro et al.^48^ show similar tendencies to those of our work, namely when the QD surrounding medium is changed from Oleic acid (OA) to perovskite (methylammonium lead iodide, MAPI), the exciton energy decreases, as occurs in the results shown in Figure 2.

### 2.2 Optical Transition Rate

**Figure 3:**
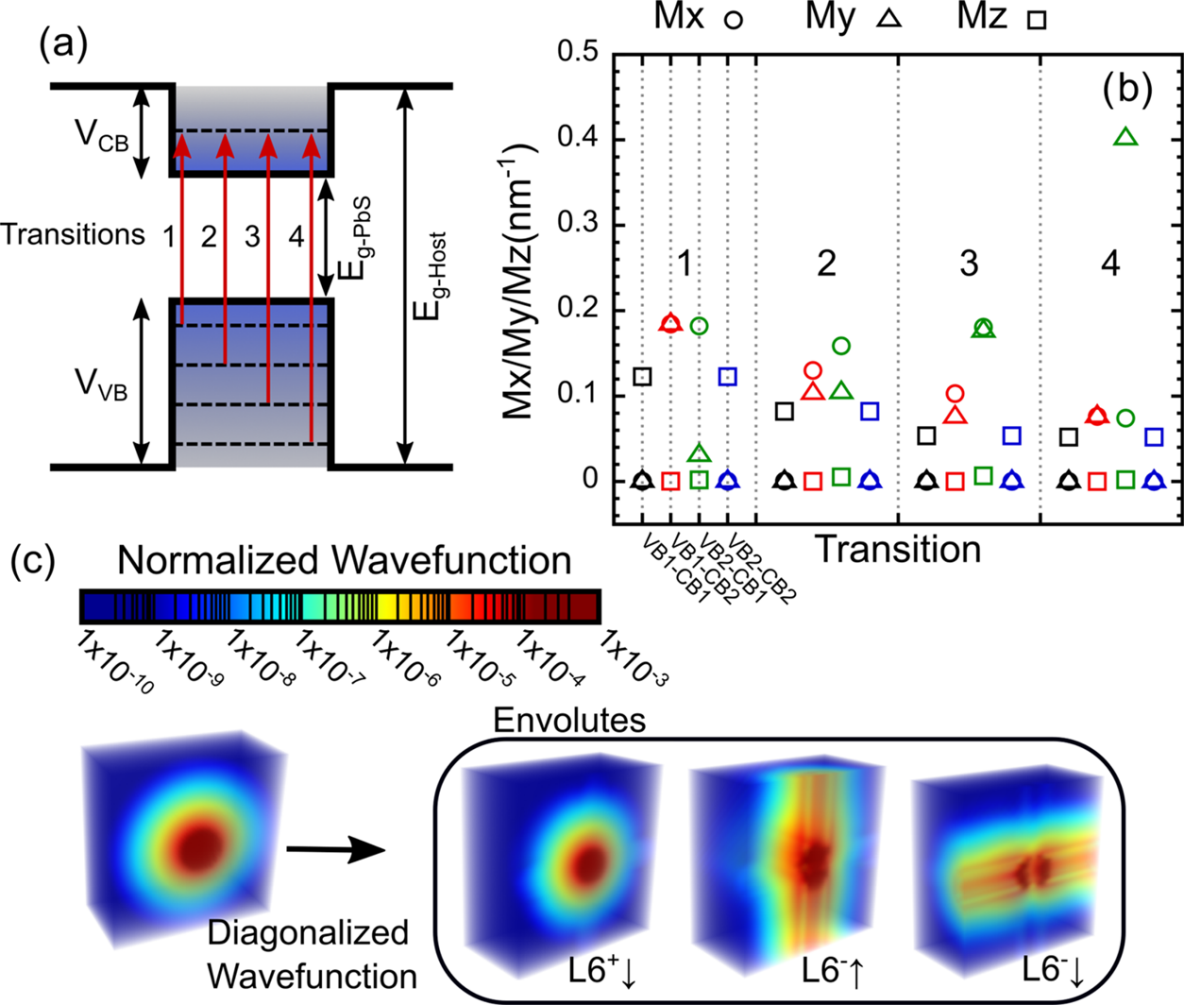
Components of the interband transitions for a 3 nm radius QD embedded in a 2.3 eV perovskite host. (a) Energy level diagram for the default case with the 4 relevant transitions calculated in panel (b) highlighted; (b) representation of all of the transition matrix elements for all of the different transitions in the default system (see Figure 1b), in which the VB and CB are doubly degenerate; (c) decomposition of the diagonalized wave function into the 4 different envelopes. The missing envelope is not shown, as it is 0.

Having determined the energy level configuration of the system, one can determine the envelope wave functions procedure detailed in the Methodology section. This process is also described graphically in Figure 3 for the default case (see Table 1), with the energy level configuration shown in Figure 3a. A QD radius of 3 nm was used in this case to guarantee the energy level in the CB Supporting Information Section S4. Figure 3c shows the conversion of the lowest CB wave function, from the diagonalized system, into the 4-band envelopes of the k.p basis (here only 3 envelopes are shown as the last one was 0). Since both VB and CB are doubly degenerate (as described in the Methodology section), each transition has 4 different band-to-band transitions (VB1-CB1, VB1-CB2, VB2-CB1, VB2-CB2), and each of those transitions has three components (*M_x_*, *M_y_*, *M_z_*) related to the polarization and incidence angle of the exciting radiation. All of these parameters are shown in Figure 3b for each of the 4 transitions in the system. The most relevant aspect here is that the VB1-CB1 and VB2-CB2 transitions behave similarly; they only show an equal magnitude *M_z_* component while *M_x_* and *M_y_* are 0. On the other hand, the VB1-CB2 and VB2-CB1 transitions show the complementary behavior as *M_z_* is always 0 while *M_x_* and *M_y_* are not. This behavior is quite interesting because the transition to the k.p basis does break the spherical symmetry; however, the separation into *z* and *x*/*y* components is reminiscent of what happens in a spherical system where there is also separation of the matrix elements into z and left/right polarization,^17,54^ which is essentially the same as *z* and *x*/*y* components. Thus, despite the basis change, some spherical behavior still remains. Beyond that, we also performed an analysis of the dependency of the transition rate on the core QD properties (effective mass, Pl, Pt, and QD radius) provided in detail in Supporting Information Section S5. We note that the QD radius has an impact on the transition rate that far outweighs all of the other factors, especially as the size increases. Nevertheless, considering that smaller sizes are preferred for many applications, the other parameters can still have a competing influence on the final transition properties. Moreover, it was also determined that the transition rate seemed to decrease with the increasing transition energy. This could be attributed to the delocalization of the wave function outside the QD, as the energy gets closer to each respective band.

Considering the high number of transitions in this system (each transition is composed of 12 independent elements), a method was devised to simplify the analysis by properly averaging all of these values. Essentially, it is based on a 2-step averaging process that reduces the granularity of an analysis of each individual element to a global analysis of the entire system, which does also get closer to a more practical context. This process starts by performing an angular average of the *M_x_*, *M_y_*, and *M_z_* values (integrating θ and φ in eq S11 of Supporting Information), followed by a standard average of the 4 different interband transitions. This then results in a single value to represent the overall transition system, *M*_avg_. The process is also shown in Figure S7 in Supporting Information Section S6.

### 2.3 QD@Host Optimization

With the transition properties calculated, it is of great interest to determine the actual properties of an effective medium that can combine the properties of both the QD and the host material, thus enabling further optical analysis and direct comparison with optical measurements. Here, it would also be possible to include recombination and charge transport effects; however, these effects are often highly dependent on the fabrication methods. Therefore, such simulations in the electrical domain are outside the scope of this work, in order to allow focus directly on the fundamental optical effects of QD@Host materials, as well as to maintain a degree of simplicity in the model and analysis to facilitate comprehensiveness and applicability.

To determine the combined optical properties of QDs and host media, a semiclassical approach was employed considering the Bruggerman effective medium formalism (detailed in the Methodology section). From there, we performed an optimization of the QD properties to determine what would be the ideal parameters for intermediate band (IB) solar cells.^11^ This is a representative example of a promising QD@Host application, in which the quantum-confined states act as intermediate mini-band(s), energetically located within the host bandgap, enabling the production of photogenerated carriers from photons with energy below host bandgap via a two-photon absorption process. For this system, it is well-known that more energy levels can negatively impact the PV performance due to thermal escape losses.^55^ Furthermore, it has also been shown that the ideal energy level configuration for maximum PV efficiency (∼50% at 1-Sun) consists in a 2.3 eV bandgap host incorporating QDs having a shallow VB and a CB with an energy level close to 0.9 eV (as depicted in Figure 1c).^39^ Taking this into account, we developed a figure of merit (FoM) that not only considers the aforementioned effects but also adds a weighting factor accounting for the overall absorption of the QD system. The FoM is defined as
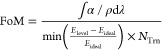
1where the term in the numerator is the integrated absorption coefficient (α) per QD density (number of QDs per unit volume, ρ), α/ρ (representing the overall absorption of the system), and the terms in the denominator are the number of transitions (*N*_Trn_) and the similarity factor, min((*E*_level_ – *E*_ideal)_)/*E*_ideal_). This factor is defined as the smallest difference between the energetic position of all of the energy levels in the CB and the ideal separation value (0.9 eV), essentially representing how close the system is to having an energy level located at the ideal IB case.

For the optimization, we chose to focus on the QD radius, the host bandgap, and the overall offset (represented in Figure 1c) between the bandgaps of the host and QD bulk materials, as these are the main parameters influencing the final absorption spectrum. The offset is a parameter defined to facilitate the calculation of the energies of the VB maximum and CB minimum for the QD bulk material, relative to the host bandgap. It is a percentage value that indicates how much of the overall bandgap energy difference, host bandgap (*E*_g-Host_), QD bulk bandgap (*E*_g-PbS_) goes to the conduction band (i.e., to *V*_CB_) and to the valence band (to *V*_VB_). For instance, an offset of 0.8 and a host bandgap of 2 eV means that the CB minimum of the QD bulk material will be at 1.28 eV [(2 – 0.4) × 0.8] and the VB maximum at 1.68 eV [(0.4 + 1.28)].

**Figure 4:**
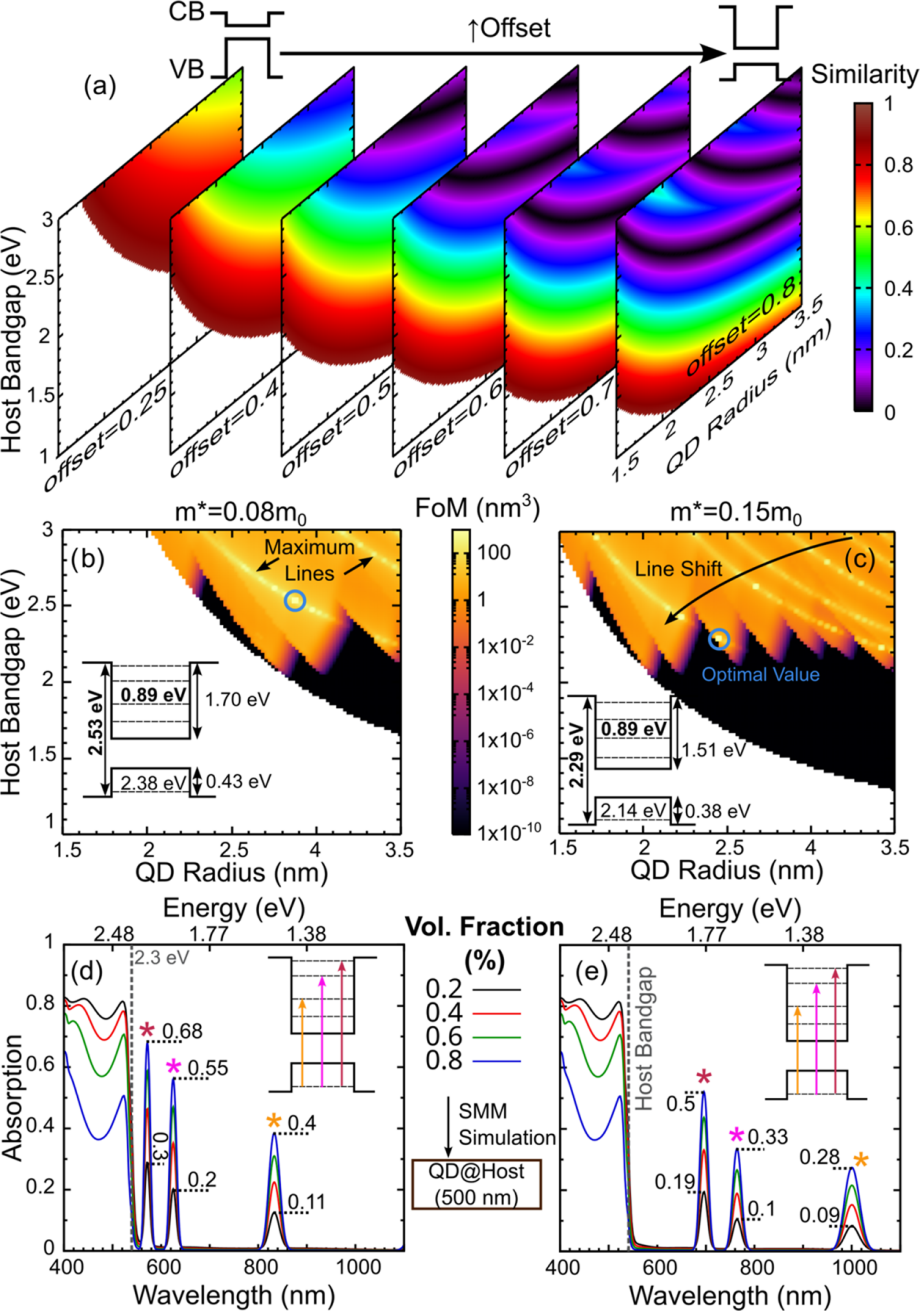
(a) Heat map plotting the dependency of the similarity factor (min((*E*_level_ – *E*_ideal)_)/*E*_ideal_)) on the host bandgap and QD radius for different band offsets (0.25, 0.4, 0.5, 0.6, 0.7, 0.8); the top schematic is a representation of the change in the band structure with the offset value. (b, c) FoM heat map results for effective masses of 0.08*m*_0_ and 0.15*m*_0_, respectively; the inset schematics represent the optimized band configuration for each case attained at the point of highest FoM marked by the blue circle in a maximum line, respectively, at QD radii of 2.8 and 2.4  nm. (d, e) Scattering Matrix Method optical simulations for a 500 nm thick single slab of the optimized QD@Host effective medium, for effective masses of 0.08*m*_0_ and 0.15*m*_0_, respectively, and for different volumetric fractions (QD vol./vol. densities in host). The inset schematics in panels (d) and (e) show the interband transitions responsible for the sub-bandgap absorption peaks marked (*) in the respective absorption spectra.

Figure 4a shows the dependency of the similarity factor (min((*E*_level_ – *E*_ideal)_)/*E*_ideal_)) on the offset value. Lower offsets (i.e., small *V*_CB_) do not get close to the targeted IB separation value of 0.9 eV, due to the shallower CB potential, and are thus of lesser relevance. As the offset increases, so does the CB depth, and several bands start appearing (black bands in Figure 4a) representing QD configurations with energy levels close to the ideal 0.9 eV value. The offset is a parameter that is determined from the QD and host band alignments which, in practice, can be changed by adapting the QD and/or host composition/doping.^18,56^ In this particular case, perovskite compounds are ideal for such considerations as it is well-known to have a highly flexible band structure.^40,57^ We chose to continue by establishing an offset value of 0.8, as Figure 4a does show several bands close to the ideal level, which can help in the FoM calculation. Furthermore, bigger offset values also lead to shallower VB which is of particular interest for IB solar cell application.

Figure 4b,c shows the FoM dependency on the QD radius and the host bandgap for different effective masses of the QD material. The effective mass values considered are 0.08*m*_0_ (the experimental PbS value)^41,42^ and 0.15*m*_0_ which is shown in Figure 2b to have a good proximity to the empirical results. Quite interestingly, the bands in the similarity profile (Figure 4a) also appear in this case and, more importantly, are responsible for defining the main regions of interest, indicated in Figure 4b as the maximum lines.

The other main factor responsible for defining the shape of FoM is the integrated absorption coefficient per density (∫α/ρdλ). Particularly for smaller host bandgaps, there seems to be a significant negative impact on the overall absorption coefficient, since below 2 eV, the absorption in the QDs is almost negligible, as seen from the near zero FoM in Figure 4b,c. As such, host bandgaps above 2 eV are preferred for this application. Other than the bandgap cutoff effect, the integrated absorption coefficient per density also indicates the parameter sets that yield highest overall light absorption. On the other hand, the number of transitions, *N*_Trn_, is mostly responsible for attenuating the overall peak intensity, which also has an effect in determining the best optimized system; however, it does not have a clear visual impact on the results.

The aforementioned factors are the three FoM terms that the optimization procedure should balance in order to output a best-case QD@Host system as close as possible to the ideal IB configuration of Figure 1c. Nevertheless, note that the FoM quantity defined in this way has units of m^3^. Dividing the FoM expression by a fourth term proportional to the QD volume would make it dimensionless. However, being the QD radius an optimization variable, that would “artificially” create a tendency for lower QD sizes, which is an effect already driven by the *N*_Trn_ variable providing a better behavior, since the IB solar cell application prefers lower transitions (ideally one) and not necessarily lower QD size. Nonetheless, as shown in Figure S9 of Supporting Information which presents results of the 0.08*m*_0_ and 0.15*m*_0_ effective masses for the dimensionless FoM, the plotted trends remain quite similar to those of Figure 4b,c.

Subsequently, we chose the optimized values for each effective mass, shown by the inset schematics in Figure 4b,c. Most interestingly, for the effective mass of 0.15*m*_0_, the best bandgap determined was 2.3 eV, which is remarkably close to the value also determined for the ideal IB solar cell energy diagram. In any case, the preferential QD@Host results are those close to the maximum lines (as indicated in Figure 4b).

### 2.4 Device Optical Analysis

As a final point, we calculated the absorption coefficient for each of the optimized cases and then proceeded to perform a complete optical analysis of a 500 nm thick QD@Host single layer, via a semianalytical Scattering Matrix Method^58,59^ (Figure 4d,e). The calculation was performed for different volumetric fractions (from 0.2 to 0.8) of QDs impregnated in the host medium, e.g., a volumetric fraction of 0 represents only host material and 1 only QD material. Figure 4d,e shows several elements in this transition where, as expected, higher fraction increases the intensity of the QD absorption peaks occurring at energies below the host bandgap and reduces the overall perovskite bulk absorption. This is a result of the lower bulk perovskite fraction (from the inclusion of QDs) coupled with possible transitions in the QDs, for energies above *E*_g-Host,_ not being accounted for. Remarkably, it is shown that even with low QD densities (e.g., 0.2 vol %), it is possible to have considerable QD-enabled absorption peaks reaching 0.3 at photon energies below the host bandgap, while with high QD densities (e.g., 0.8 vol %), the below-bandgap absorption can reach peak values close to 0.7. Furthermore, it is also clear that the overall peak intensity increase with volume fraction is roughly linear, as most peaks have an absolute increase of ∼0.3 absorption from fractions of 0.2–0.8.

It should also be noted that the simplistic Bruggerman effective medium approach taken here does not consider factors such as the actual PbS bulk absorption, or other perturbative effects on the energy levels, that can both lead to a absorption peak broadening and an overall background absorption as reported experimentally.^18^ These effects, if properly accounted, would raise the overall absorption of the QD@Host film for energies above *E*_g-Host_, and thereby counteract the above-bandgap perovskite absorption losses seen in the results of Figure 4d,e.

## 3 Conclusions

In this work, we present a detailed semiclassical approach to accurately compute the optical properties of nanostructured QD@Host materials, based in a 4-band k.p method to determine the absorption coefficient and a Bruggerman effective medium formalism to predict the complex refractive index with arbitrary density of impregnated QDs. Although the calculations were focused on PbS@Perovskite in view of this promising material combination for photovoltaics, the methodology can be readily applied to any rock-salt QD material and any host semiconductor.

After validation of the model against empirical results, the transition properties between the several interband elements of the system were calculated. Then, we performed an optimization, focused on intermediate band (IB) solar cell application, where the main figure of merit factors used was the number of transitions, the absorption coefficient per density, and the closeness of the energetic position of the quantum-confined level(s) to the ideal IB energy diagram. From the optimized results, we then performed a simple optical device analysis of the final effective medium that combined the properties of both the QDs and the host material. From this analysis, it was determined that the QDs inclusion can pronouncedly benefit the overall optical performance of the semiconductor system, resulting in a much broader photoresponse spectrum with high levels of light absorption at photon energies below the host bandgap energy.

## 4 Methodology

In this work, we developed a 4-band envelope function model based on an empiric k.p Hamiltonian^60^ for rock-salt semiconductors.^36^

This method consists of a three-step approach. First, we assume the eigenvalues follow parabolic shapes with the experimental effective masses to guarantee that the results are experimentally consistent. It is thus possible to build a diagonal Hamiltonian that takes implicitly into account spin–orbit coupling and the crystal strain.^60,61^ This diagonal Hamiltonian (eq 2) can then be solved to obtain the eigensolutions for the different bands (*E*_band_ and *E* signs in eq 2 depend on the band positive for CB and negative for VB). More importantly, it is possible to adjust the energy configuration of the system, such that the diagonalized Hamiltonian gets developed into an effective mass one-band model, already studied previously in many reports for different QD shapes.^17,62,63^ For this work, we used a spherical step-like potential, as it is the closest shape to the CQD,^64−66^ similar to a previous work.^17^
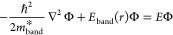
2Here, ℏ is the reduced Planck’s constant, m*_band_ is the effective mass for the specific band, Φ is the wave function, *E*_band_ is the potential for the band (in this case is a standard spherical step-like potential), and *E* is the eigenenergy.

In the second step, we define the diagonalization matrix that restores the diagonalized eigenfunctions to their initial basis by using a 4-band k.p Hamiltonian (eq 3) for rock-salt materials.^36,67^ This essentially builds the envelope functions, Ψ_ν_, that represent the nanostructure in the k.p method. For simplicity, and due to the similarity of CB and VB effective masses for common lead-salts,^36^ we chose *m*_l_ = *m*_t_ = *m* (a global effective mass). The diagonalization matrix is then created from the eigenvectors of the k.p Hamiltonian, process is fully detailed in Supporting Information Section S1.
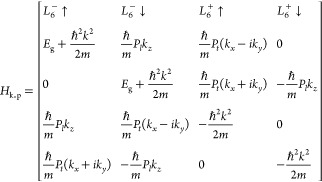
3

Here, *E*_g_ is the host material bandgap, *k*_*x*_, *k*_*y*_, and *k*_*z*_ are the *x*, *y*, and *z* components of the wave-vector in the reciprocal space, respectively, *k* is the wave-vector magnitude, Pl and Pt are the longitudinal and transverse momentum matrix elements, and *L*_6_^(±)^(↑/↓) are the different *L* points in the k.p model.

The envelope functions can be expanded as a series of plane waves.^60^ As such, it is possible to connect the diagonalized eigenfunctions to the envelope functions through discrete Fourier transform (DFT), since the transformation matrix is applied in the wave-vector domain. Thence, the envelope functions can be determined by first applying the DFT to the diagonalized eigenfunctions, then changing basis with the transformation matrix, and finally reverting to the space domain by applying the DFT. The conversion is detailed in Supporting Information Section S1.

Lastly, using the envelope functions, it is possible to determine the transition rate for different interband transitions. The dipole matrix element, ⟨Ψ|ϵ·*r*|Ψ′⟩, for the photon-electron interaction is the sum of the dipole elements of the different envelope functions *L*_6_^(±)^(↑/↓). Where, **ε** is the light polarization vector and **r** the position vector
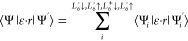
4Lastly, the absorption coefficient can be determined from the matrix elements. Here, we chose to calculate the absorption coefficient per density (α/ρ [nm^3^/cm] eq 5) as it extracts the QD density dependence that can easily be added after the calculations to determine the final set of observable properties.
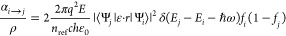
5where *q* is the electron charge, *E* is the transition energy, *n*_ref_ is the host refractive index, *c* is the speed of light in vacuum, *h* is Planck’s constant, ε_0_ is the permittivity of free space, *E_j_* and *E_i_* are the final and initial energies, respectively, and δ is the delta function that represents conservation of energy for the transition. This latter parameter is approximated via a Gaussian curve, where the standard deviation represents the QD dispersion.^17,60^ The *f_i_* and *f*_f_ are the filling factors that indicate how full/empty each state is. For simplicity, we considered the initial state to be completely full and the final state to be completely empty (*f_i_* = 1 and *f_j_* = 0). The factor of 2 multiplying the expression in eq 5 accounts for the spin degeneracy of each state.

After the overall absorption coefficient for the system is determined, it is important to assess the measurable optical properties of the nanostructured medium that combines the physical properties of host and QD material, thus effectively describing the QD@Host material from an optical standpoint. For that, the Bruggerman effective medium approach (eq 6) was chosen:
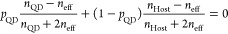
6where, *p*_QD_ represents the volumetric fraction (vol./vol.) of QDs immersed in the host material, and *n*_QD_, *n*_Host_, and *n*_eff_ represent the complex refractive index of the QD, the perovskite material, and the effective medium, respectively. From the effective medium results (*n*_eff_), it is then possible to study the overall optical properties of the system.

Lastly, we note that this semiclassical effective medium approach leads to the double accounting of the QD volumetric fraction both in the Bruggerman equation and in the α/ρ factor of eq 5. To circumvent this issue, the absorption coefficient has been calculated taking the highest possible density of QDs, corresponding to a hexagonal closed-packed lattice, while the actual volumetric density is considered only in the Bruggerman equation.

All of the code used to obtain the results provided in this work is available online.^68^

## Supporting Information Available

(1) Mathematical foundation, for the model shown in the manuscript; (2) finite elements method (FEM) development of the Schrödinger equation, necessary for the analysis in Figure 2; (3) a study of the QD bandgap in function of the Host bandgap; (4) convergence testing of the 4-band k.p model results; (5) dependency of the QD intrinsic properties on the convergence parameters; (6) detailed description of the process to average all of the transition elements for a single transition; (7) the optical properties (complex refractive index) of the materials used in the simulations; (8) description of the optimization results considering a nondimensional Figure of Merit (PDF)
