# Supplementary material for: Understanding the Potential of Light Absorption in Dots-in-Host Semiconductors
Source: ACS Photonics. 2024 Jul 24;11(10):4048–57. doi: 10.1021/acsphotonics.4c00760 (PMC11487685; doi:10.1021/acsphotonics.4c00760)
Supplement: Supplementary file 1 — ph4c00760_si_001.pdf [file ph4c00760_si_001.pdf]

Supplementary Material

Understanding the Potential of Light Absorption in Dots-in-Host Semiconductors

Miguel Alexandre*, Hugo Águas, Elvira Fortunato, Rodrigo Martins, Manuel J. Mendes*

i3N/CENIMAT, Department of Materials Science, NOVA School of Science and Technology and CEMOP/UNINOVA, Campus de Caparica, 2829-516 Caparica Portugal

Corresponding Authors: m.alexandre@campus.fct.unl.pt, mj.mendes@fct.unl.pt

S1. Mathematical Foundations

Here the authors provide a detailed development of the theoretical concepts used to obtain the results shown in the Main Manuscript. The complete development of Equation 1 in the Main Manuscript was already shown in a previous work.[1]

The diagonalization matrix can be created via the eigenvectors of the k.p Hamiltonian shown in Equation 3 in the Main Manuscript. To determine the eigenvectors, it is first necessary to determine the eigenvalues, that are as follows (each eigenvalue is doubly degenerate):

$$E_{L_6^- \uparrow/\downarrow} = E_g + \frac{\hbar^2 k^2}{2m} - \frac{E_g}{2} - \sqrt{\left(\frac{E_g}{2} + \frac{\hbar^2 k^2}{2m}\right)^2 + (A^2 + |B|^2)} \quad \text{(S1)}$$

$$E_{L_6^+ \uparrow/\downarrow} = -\frac{\hbar^2 k^2}{2m} - \frac{E_g}{2} - \sqrt{\left(\frac{E_g}{2} + \frac{\hbar^2 k^2}{2m}\right)^2 + (A^2 + |B|^2)} \quad \text{(S2)}$$

Where E_g is the host bandgap, m is the effective mass of the QD material, k^2 is the wave-vector magnitude, $A = \hbar P_l k_z / m$ and $B = \hbar P_t (k_x - i k_y)/m$. In these last simplifications, P_l and P_t are the longitudinal and transverse momentum matrix elements.

The eigenvectors are thus as follows.

$$|L_6^- \uparrow\rangle = \frac{1}{C_\alpha}\begin{bmatrix} -B \\ A \\ 0 \\ \alpha - \sqrt{\alpha^2 + A^2 + |B|^2} \end{bmatrix} \quad \text{(S3)}$$

$$|L_6^- \downarrow\rangle = \frac{1}{C_\alpha}\begin{bmatrix} A \\ B^* \\ -\left(\alpha - \sqrt{\alpha^2 + A^2 + |B|^2}\right) \\ 0 \end{bmatrix} \quad \text{(S4)}$$

$$|L_6^+ \uparrow\rangle = \frac{1}{C_\alpha}\begin{bmatrix} 0 \\ \alpha - \sqrt{\alpha^2 + A^2 + |B|^2} \\ B \\ -A \end{bmatrix} \quad \text{(S5)}$$

$$|L_6^+ \downarrow\rangle = \frac{1}{C_\alpha}\begin{bmatrix} \alpha - \sqrt{\alpha^2 + A^2 + |B|^2} \\ 0 \\ A \\ B \end{bmatrix} \quad \text{(S6)}$$

Here, the authors chose the following simplifications:

$$\alpha = \frac{E_g}{2} + \frac{\hbar^2 k^2}{2m}$$
$$C_\alpha = \sqrt{\left(\alpha - \sqrt{\alpha^2 + A^2 + |B|^2}\right)^2 + A^2 + |B|^2} \quad \text{(S7)}$$

The adjoint transformation matrix is necessary for the similarity transformation as it is the matrix that expands the diagonalized eigenfunctions in the k.p basis (Equation S8 - top equation for $L_6^+ \uparrow$). It is important to note that the diagonalized solutions are a column vector with a single unitary entry — Equation S8 bottom equation — representing each band (in this case there are two degenerate conduction and valence bands, as required by the k.p Hamiltonian).

$$\Psi_{|L_6^+\uparrow\rangle} = \sum_{i}^{CB_1,CB_2,VB_1,VB_2} \langle L_6^+\uparrow|\Phi_i$$

$$|CB_1\rangle = \begin{bmatrix}1\\0\\0\\0\end{bmatrix}\Phi_{CB_1}, |CB_2\rangle = \begin{bmatrix}0\\1\\0\\0\end{bmatrix}\Phi_{CB_2}, |VB_1\rangle = \begin{bmatrix}0\\0\\1\\0\end{bmatrix}\Phi_{VB_1}, |VB_2\rangle = \begin{bmatrix}0\\0\\0\\1\end{bmatrix}\Phi_{VB_2} \quad \text{(S8)}$$

As such, the transformation matrix can be defined as follows (Equation S9), where each element represents one of the expansion coefficients of the envolute function in terms of the diagonalized functions.

$$T^+ = \begin{bmatrix}\langle L_6^-\uparrow|\\ \langle L_6^-\downarrow|\\ \langle L_6^+\uparrow|\\ \langle L_6^+\downarrow|\end{bmatrix} = \frac{1}{C_\alpha}\begin{bmatrix}-B^* & A & 0 & \beta\\ A & B & -\beta & 0\\ 0 & \beta & B^* & A\\ \beta & 0 & A & B^*\end{bmatrix} \quad \text{(S9)}$$

$$\beta = \alpha - \sqrt{\alpha^2 + A^2 + |B|^2}$$

Having determined the transformation matrix, it is then possible to convert the diagonalized eigenfunctions into the k.p envolutes, as described in the Main Manuscript.

The final absorption coefficient can be determined from the Main Manuscript Equation 5. Where, the transition elements can be calculated via Equation 4. Here, it is often useful to decompose the polarization angle in terms of its Euler angles (θ, φ). Thus,

$$\begin{aligned}\langle\Psi|\epsilon\cdot r|\Psi'\rangle &= cos(\phi)sin(\theta)\langle\Psi|x|\Psi'\rangle\\ &+ sin(\phi)sin(\theta)\langle\Psi|y|\Psi'\rangle\\ &+ cos(\theta)\langle\Psi|z|\Psi'\rangle\end{aligned} \quad \text{(S10)}$$

Notably, as explored in the Results Section 2, to reduce the number of variables, the authors performed a global angular average of the matrix elements (integration from [0, 2π] for φ and [0, π] for θ). Equation S11 shows how to perform the angular averaging process.

$$\begin{aligned}\left|\langle\Psi_j|\epsilon\cdot r|\Psi_i\rangle\right|^2 &= cos^2(\phi)sin^2(\theta)\left|\langle\Psi_j|x|\Psi_i\rangle\right|^2\\ &+ sin^2(\phi)sin^2(\theta)\left|\langle\Psi_j|y|\Psi_i\rangle\right|^2\\ &+ cos^2(\theta)\left|\langle\Psi_j|z|\Psi_i\rangle\right|^2\\ &+ 2cos(\phi)sin(\phi)sin(\theta)\mathrm{Re}[\langle\Psi_j|x|\Psi_i\rangle\langle\Psi_j|y|\Psi_i\rangle\\ &+ 2cos(\phi)sin(\theta)cos(\theta)\mathrm{Re}[\langle\Psi_j|x|\Psi_i\rangle\langle\Psi_j|z|\Psi_i\rangle\\ &+ 2sin(\phi)sin(\theta)cos(\theta)\mathrm{Re}[\langle\Psi_j|y|\Psi_i\rangle\langle\Psi_j|z|\Psi_i\rangle\end{aligned} \quad \text{(S11)}$$

$$\left|\langle\Psi_j|\epsilon\cdot r|\Psi_i\rangle\right|^2_{\langle\phi_{avg}\rangle\langle\theta_{avg}\rangle} = \frac{\pi}{4}\left(2\left|\langle\Psi_j|x|\Psi_i\rangle\right|^2 + 2\left|\langle\Psi_j|y|\Psi_i\rangle\right|^2 + \left|\langle\Psi_j|z\right|\right.$$

At this point, there is an angular averaged matrix element for each band transition (VB1→CB1, VB1→CB2, VB2→CB1, VB2→CB2). The average of these 4 transitions is taken to obtain the final value. This process is also elaborated graphically in Figure S7.

The theoretical developments were then implemented and developed in Python®. The code is also available in a Github® repository (reference [2]).

S2. FEM Development of the Schrödinger Equation

Figure 2 of the Main Manuscript shows the results of several finite elements method (FEM) simulations, made to complement the standard analytical results from the spherical well

potential. In this section we describe the mathematics behind this process. The authors follow the description shown in the Deall.II tutorial library, exercise 36.[3]

In FEM simulations, the first objective is to convert the differential equation into the weak form, such that it is then possible to build the matrix–vector equation that will be used to solve the problem. Here, the problem is defined by the Schrödinger equation (Equation S12 in non-dimensionalized form), where V(x) is the acting potential and Ω and ∂Ω represent the simulation domain and boundary, respectively. As numerical simulations always require boundary conditions — to define the simulation domain — the standard choice of Dirichlet boundary conditions was made. In this Dirichlet condition it is fundamental to consider a simulation region much larger than the QD size, in order to guarantee that the wavefunction, Ψ, has vanished in the boundaries (essentially confirming the boundary condition).

$$\begin{aligned} [-\Delta + V(x)]\Psi(x) &= E\Psi(x) && \text{in } \Omega\,, \\ \Psi &= 0 && \text{on } \partial\Omega\,. \end{aligned} \quad \text{(S12)}$$

Here, the standard finite-element approach is used by multiplying the equation from the left by a test function, integrating and then considering the wavefunctions expanded in a finite dimensional space $\Psi(x) \approx \Psi_h(x) = \sum_j \phi_j(x)\tilde{\psi}_j$. The resulting discretized eigenvalue problem is then as follows (the bottom equation is in matrix-vector notation).

$$\sum_j \left[\int_\Omega \nabla\phi_i \nabla\phi_j + \int_\Omega V(x)\phi_i\phi_j \right] \tilde{\psi}_j = E_h \sum_j \left[\int_\Omega \phi_i\phi_j \right] \tilde{\psi}_j$$
$$A\tilde{\Psi} = E_h M \tilde{\Psi} \quad \text{(S13)}$$

Here, A is the stiffness matrix and M the mass matrix.

The Deal.II library[3] was used to solve the problem. This library provides all the necessary structures to build and solve the problem (create grid, implement boundary conditions, solve the eigenvalue problem). In this case, the local integrals (built from the test functions) were solved using a 2nd order Gaussian quadrature. The eigenvalue problem was then solved using the Deal.II wrapper for the SLEPc Krylov-Schur eigenspectrum Solver.

The first process in the simulation focused on determining the accuracy and the mesh requirements needed to achieve the intended results. For that, the authors started by solving a reference spherical well problem, thus facilitating the comparison between the analytically obtained and numerically obtained results. Figure S1 shows the results for different meshes (the different vertical sections are labelled from 1–6 according to the mesh refinement) and simulation size (colour of the dots in each profile). The QD properties used for these simulations are V = 1 eV, m = $0.1m_0$ and QD radius of 3.5 nm.

As a rule of thumb, global mesh refinements bellow 3 do not seem to provide accurate results. Beyond 3, there is still a clear dependency on the simulation size (bigger simulation size does require more refinement steps to better resolve QD features). Furthermore, as the

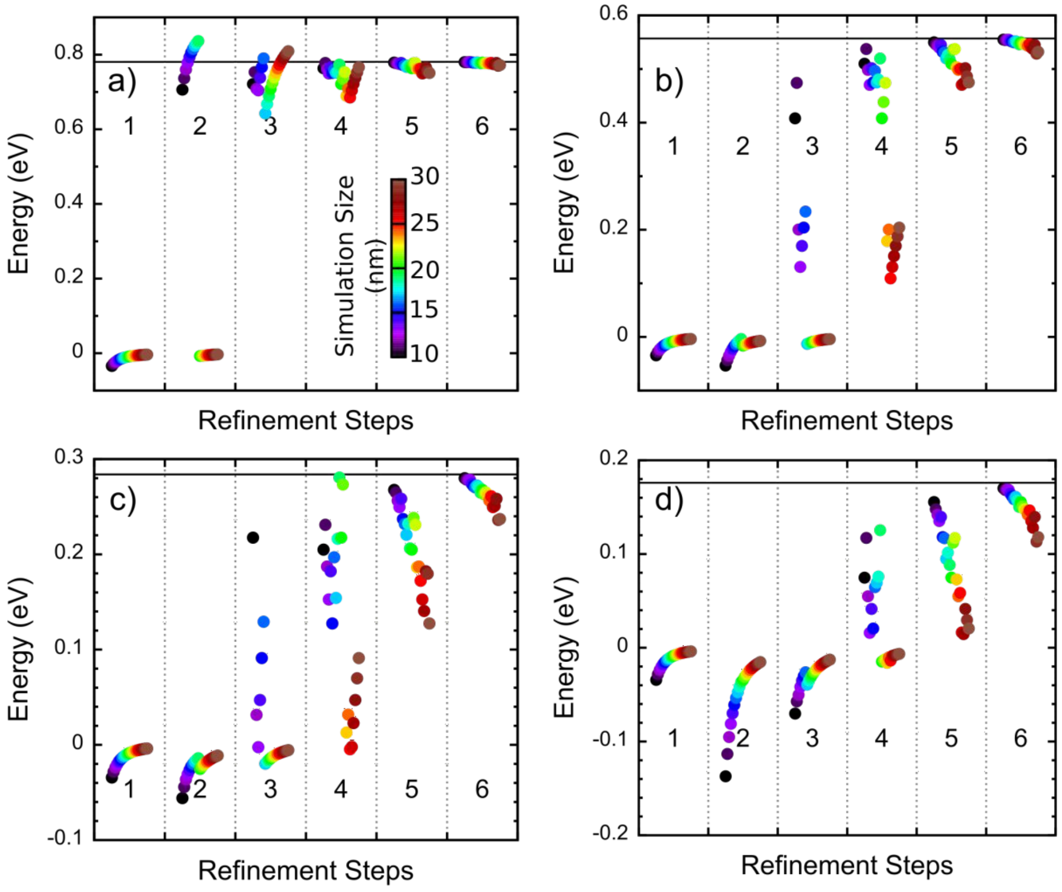

Figure S1: Convergence results for the FEM analysis of the spherical well problem. The top horizontal line represents the analytical solution. Each profile is subdivided into 6 different regions (for each refinement step in the algorithm) and in each region multiple simulation regions were tested (shown by different colour dots). The results are shown for a) first energy level; b) second energy level; c) third energy level; d) fourth energy level.

energy levels increase there is also a tendency for the error to increase. This could be from the increasing delocalization of the wavefunction as the energy level increases. For the Main Manuscript results, the authors only used the first energy level. As such, a global mesh refinement of 5 was used, as Figure S1 a) clearly shows this mesh to be independent of the simulation size and thus the ratio QD Size/Simulation Size.

A relevant aspect of the FEM simulations is the ability to more easily adapt the function representing the potential well, as the calculations are numerical and not analytical. For that, we provided in the Main Manuscript an analysis of the QD Bandgap with changing steepness of the potential (Figure 2 a)), using a standard error function

$$erf(z) = \frac{2}{\sqrt{\pi}} \int_0^z e^{-t^2}\, dr \qquad \text{(S14)}$$

We performed a change of variable of $z \rightarrow kx$, where k indicates an offset on the overall integration of the exponential. For values lower than 1, the integration will happen slowly and thus the function has a slower change in space (inset profiles of Figure 2 a) in the Main Manuscript). This k variable has been termed the function **steepness,** as it represents the rate of change of the potential in space.

S3. Bandgap Studies

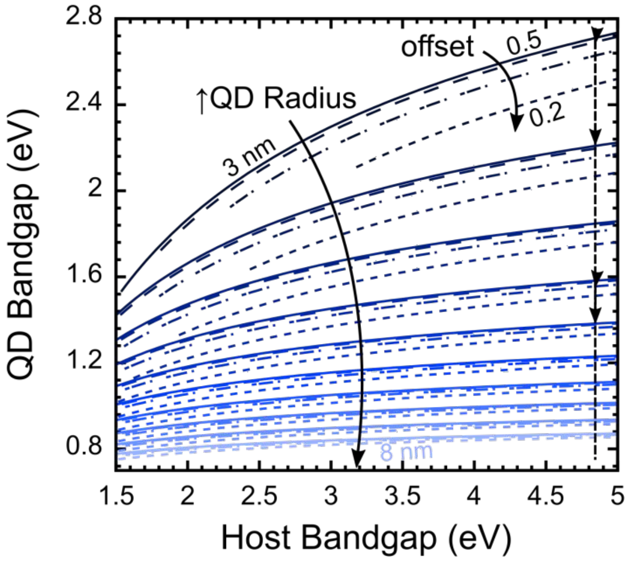

Figure S2: Quantum Dot Bandgap dependency on the Host Bandgap, QD radius (from 3 to 8 nm for different coloured lines, in steps of 1 nm) and Potential offset (0.5, 0.4, 0.3 and 0.2, as represented in Figure 1 c), from the solid, dashed, dot dashed and small dash lines, respectively).

Figure S2 summarizes the dependency of the QD bandgap ($E_{g\text{-}QD}$) on different factors. Firstly, the QD radius, expectedly, shows a decrease in $E_{g\text{-}QD}$ as the size increases, with the arrows on the right side of the plot indicating an exponential-like behaviour, also clearly seen in Figure 2 of the Main Manuscript. The Host Bandgap ($E_{g\text{-}Host}$) shows a more pronounced influence for lower QD sizes. As the QD size increases the energy levels tend to become more static (less influenced by size/potential changes). As the Host Bandgap increases there is a general trend to increase the QD bandgap, which conforms with the idea that, in the limit of the host bandgap tending to infinity, the results should tend to the infinite well problem, as marked in Figure 2 in the Main Manuscript. There is also an influence of the potential offset (difference between the potential height in the CB and VB, as defined in Figure 1 c)) on the QD bandgap. Offset values bellow 0.5 (bandgaps alignment corresponding to $V_{CB}=V_{VB}$) essentially shift the results down as represented in Figure S2.

S4. Convergence Tests

This section has 2 different parts. Firstly, the authors provide a heat map plot that describes the number of energy levels present in a single band of the QD system (Figure S3), depending on the size of the potential barrier and on the radius of the QD. This is important as it defines a good lower limit for the QD radius during the simulations, since at least one level is needed in the QD CB to calculate the interband transition properties. From the plot it can be seen that 3 nm is a minimum radius that can be used independently of the potential barrier.

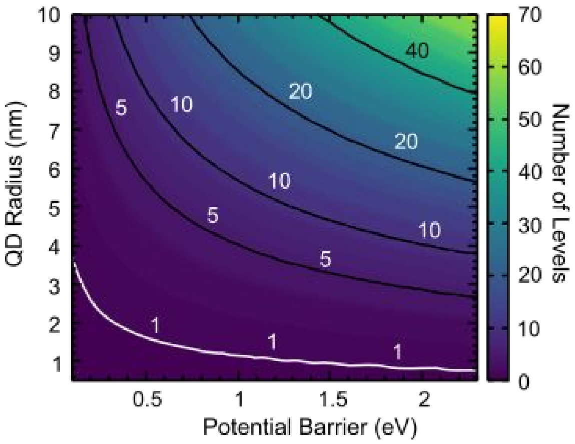

Figure S3: Heat map plot that indicates the number of energy levels present in a single band of the QD system, for changing QD radius and potential barrier. At least one level (marked by the white contour) is necessary in order to allow calculating interband transitions.

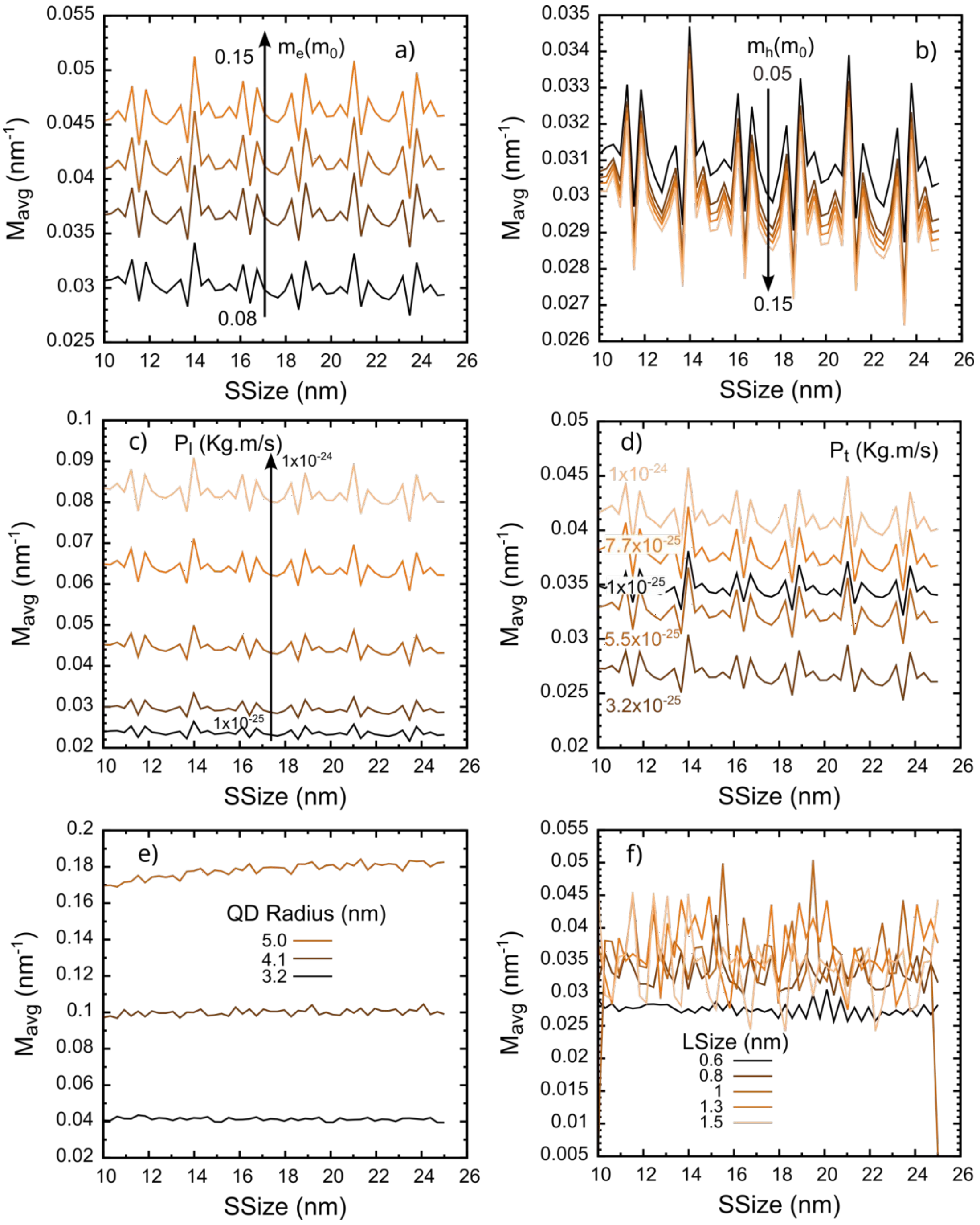

Figure S4: Convergence tests for the simulation size (SSize) dependency. a), b) dependency for changes in the electron and holes effective masses, respectively; c), d) changes on the longitudinal (P_l) and transverse (P_t) momentum matrix elements, respectively; e) dependency on the QD radius and f) dependency on the lattice size.

The second set of results show the dependency of the several parameters on the simulations size (SSize - Figure S4) and lattice size (LSize - Figure S5). The objective here is to determine the best simulation conditions that provide the best accuracy. The results do show that the simulation size, even though with some noise, does not have a significant impact on the results. Nevertheless, it is clear from the interaction with the lattice size, that lower values of the latter are preferred. From the lattice size results, it is also clear that lower values are preferred, as the results do show a general trend to increase with increased LSize.

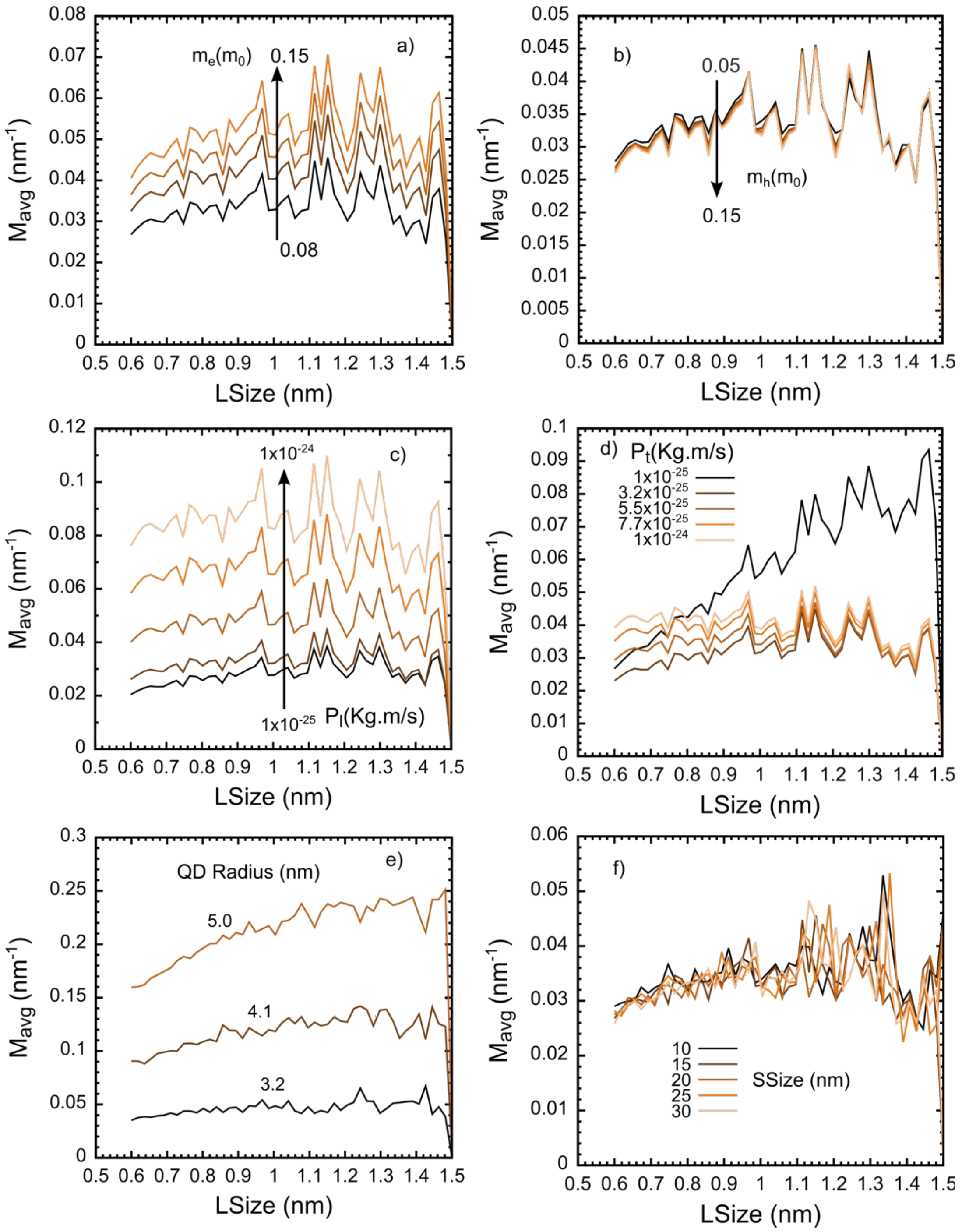

Figure S5: Convergence tests for the lattice size (LSize) dependency. a), b) dependency for changes in the electron and holes effective masses, respectively; c), d) changes on the longitudinal and transverse momentum matrix elements, respectively; e) dependency on the QD radius and f) dependency on the simulation size.

Furthermore, the actual parameters also have little impact on the results, as for the most part, changing these parameters simply moves the curves upwards or downwards, which is a consequence of the actual effect of the parameter rather than lower accuracy in the calculations.

S5. QD Properties Dependency on Convergence Parameters

This Section summarizes the transition rate properties (presenting M_{avg}) for the different relevant parameters of the system (effective mass, a) and c), Pl b), Pt d) and QD radius e)). It also shows a schematic of the simulation grid used for the process (Figure S6 f)). The grid is relevant during the basis change, namely, to calculate the discrete Fourier transform (DFT). We defined two main variables for the grid, the simulation region (SSim) and the lattice size (LSize), that describe the resolution of the calculation. Ideally, the lattice size should be as small as possible, and conversely for the simulation size. However, the lattice size is limited to the actual lattice size of the QD material (~0.6 nm for PbS [4]) due to the intrinsic properties of the method.[5] Furthermore, increasing the simulation size also increases the number of cells in the simulation region, and thus the complexity/time of calculation. As such, the authors chose to perform the calculation with different lattice sizes (0.6 and 0.8 nm) and simulation sizes (15 and 25 nm), to probe how these factors influence the results. Section S4 also has more results on the influence of these factors.

Although there is some dependency on the grid parameters (LSize and SSize), the overall trend for each parameter is the same. Furthermore, the actual differences from grid adjustment are relatively small, with the most diverging cases happening for larger QDs, that are also of less interest and where distinction between bulk and quantum behaviour becomes more complex (as also discussed for Figure 2 in the Main Manuscript). In terms of the actual transition rate dependency, the QD radius far outweighs all the other factors, especially as the size increases. Smaller sizes — that can be preferable to many applications in view of larger level separation — follow this trend as well, however the other properties also start having a competing influence. Interestingly, there seems to be a general trend of reducing the transition rate for higher energy transitions (i.e. for higher transition numbers). That could be attributed to the de-localization of the wavefunction outside the QD, as the energy levels get closer to each respective band.

The QD size influence shows a cut-off for values under 2.9 nm. This is related with the shallow CB potential used (0.4 eV) that only allows for energy levels beyond this point (shown in Figure S3). This is a limitation of the model, where certain conditions (such as shallow potential and smaller sizes) can lead to non-existent energy levels in the system. Nevertheless, lower asymmetry in the band offset (defined in Figure 1 c) of the Main Manuscript) for the CB and VB would allow for transitions in significantly smaller QDs — as can be seen in Figure S2. Such changes, however, may be detrimental to several applications as they can promote thermal losses.[6,7]

Lastly, Figure S6 a), c) and e) also show an interesting noisy behaviour. This is a consequence of the averaging of the several sub-transitions (band-to-band and Mx, My, Mz), and is particularly noticeable in the QD size as it is the most impactful parameter for the energy level configuration. Essentially, to minimize error propagation from lower probability transitions (for instance $Mx < 1x10^{-4}$) we considered such values to be 0. However, as the properties change so do these values, such that, passing this threshold can add bumps in the overall profile. Furthermore, this effect can be compounded when multiple transition elements become significant concurrently.

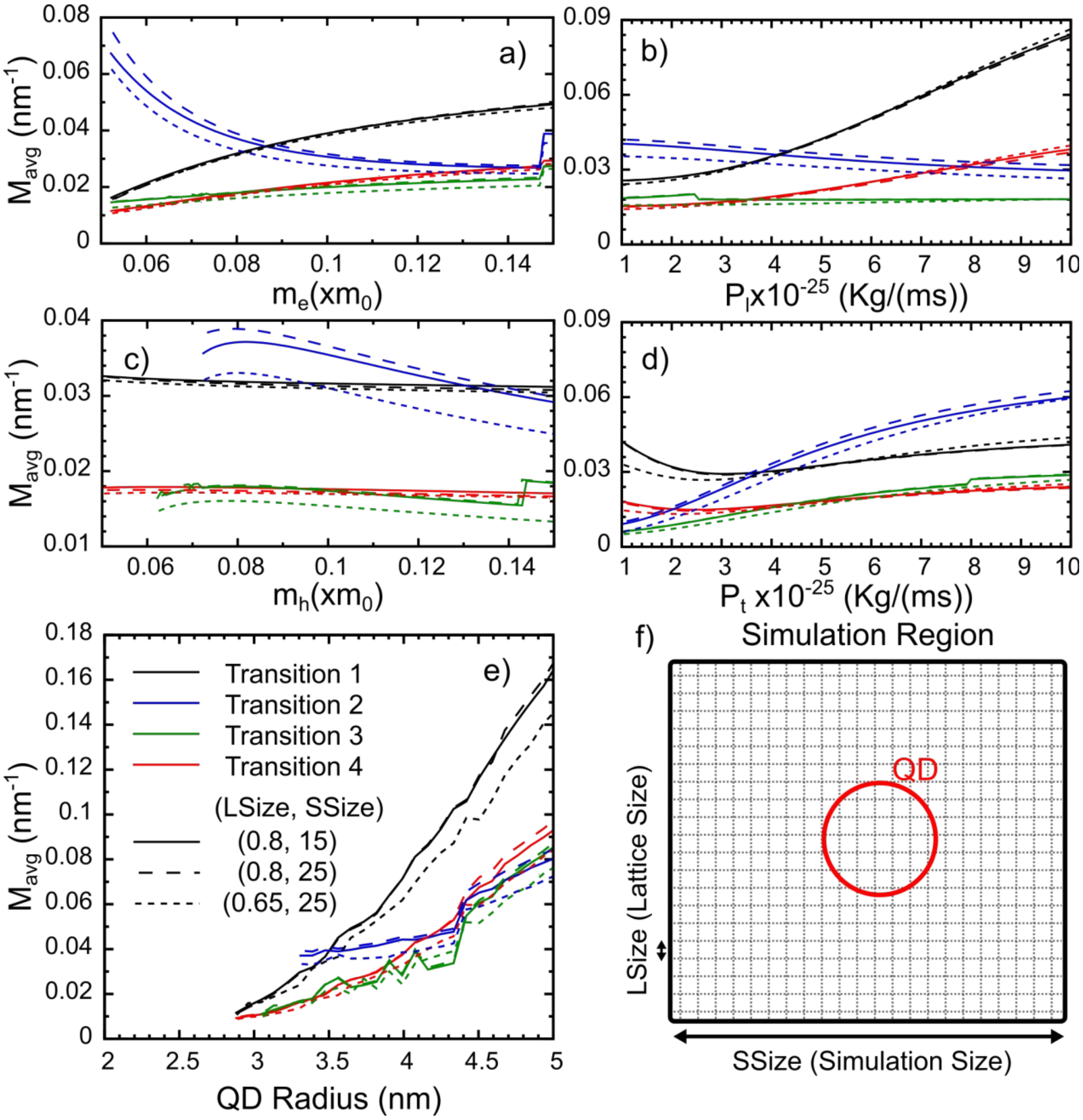

Figure S6: Transition rate (M_{avg}) dependency on the different relevant properties under study, for the four possible transitions (black, blue, green, red lines, as in **Error! Reference source not found.** a) of the Main Manuscript) and 3 combinations of lattice size (Lsize) and simulation size (Ssize) (0.8/15 nm, 0.8/25 nm, 0.65/25 nm) — full line, long dash and short dash, respectively, as a function of: a) electron effective mass (m_e), b) longitudinal momentum matrix element (P_l), c) hole effective mass (m_h), d) transverse momentum matrix element (P_t), e) QD radius. f) Schematic of the simulation grid used to perform the change of basis, emphasizing the most important elements (LSize and SSize).

S6. Averaging Transitions

This section describes graphically the 2-step process used to average the 12 elements (combination of light polarization/incidence elements and band-to-band transitions) obtained from the transition properties. The process starts with the 12 elements, represented by the dashed lines, that are then angularly averaged obtaining 4 different results (solid lines). These results are then averaged again to obtain a final value that describes the entire problem (black line).

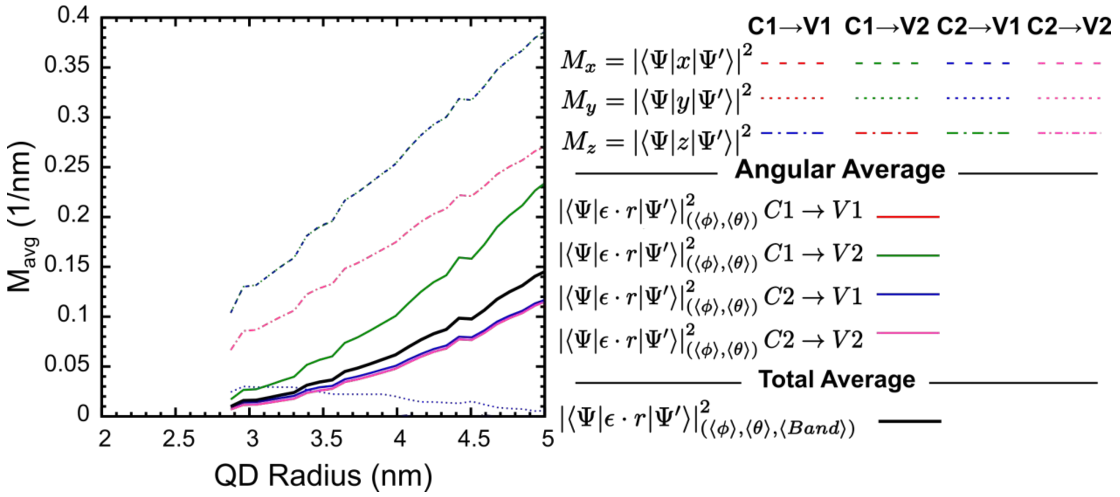

Figure S7: Graphical representation of the 2-step averaging process of the transition rates.

S7. Optical properties

In this section, the authors provide the bulk refractive index spectra, obtained from literature, for the wide-bandgap perovskite [8] and PbS [4] materials employed in the calculation of the effective medium, following the Bruggerman formalism presented in the Main Manuscript.

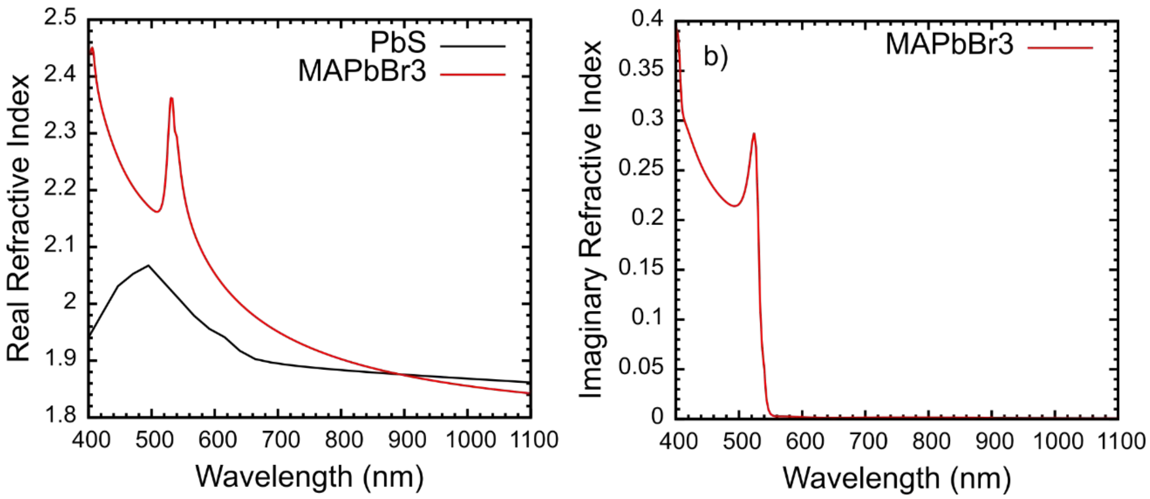

Figure S8: Optical properties taken for the constituent bulk materials of the QD (PbS) and Host (wide-bandgap perovskite, MAPbBr3) used for the Bruggerman effective medium calculations.

S8. Non-Dimensional Figure of Merit

Here, the authors provide the results for the dimensionless FoM (Figure S9) as calculated from the following equation.

$$FoM = \frac{\int \alpha/\rho \, d\lambda}{min\left(\frac{E_{level} - E_{ideal}}{E_{ideal}}\right) \times N_{Trn} \times QD_{radius}^3} \quad \text{(S13)}$$

The setup is the same as the that of the Main Manuscript Equation 1, but with the added QD_{radius} factor cubed that guarantees the non-dimensionality of the FoM.

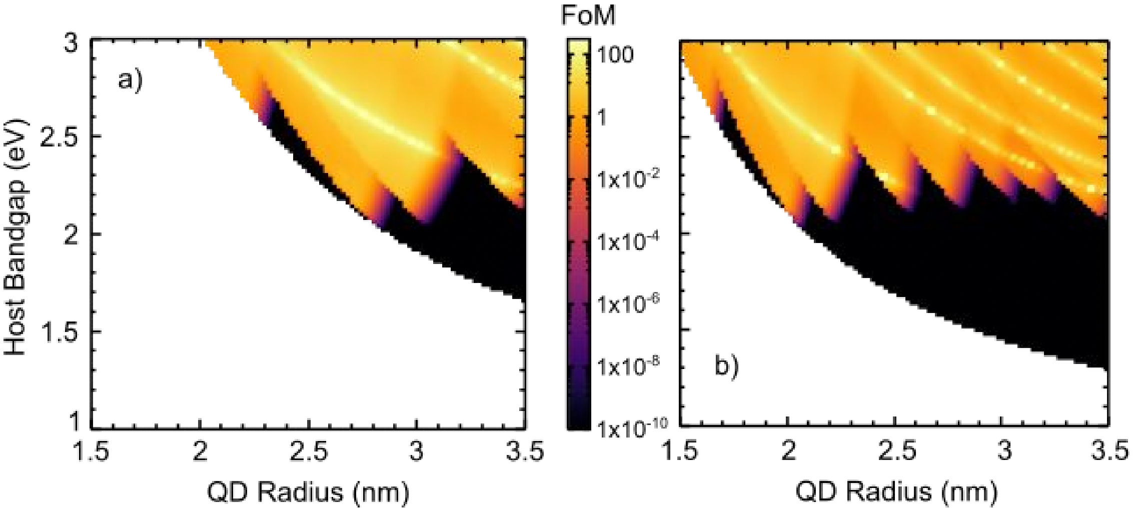

Figure S9: Non-dimensional FoM calculated from Equation S13, for a QD effective mass of a) $0.08m_0$ and b) $0.15m_0$.

As stated in the Main Manuscript, the results are quite similar to those of Figure 4, with the slight reduction in the overall peak intensity, due to the extra factor in the denominator. Regardless, the added effect does not significantly influence the results already provided in the Main Manuscript.

References

[1] M. Alexandre, H. Águas, E. Fortunato, R. Martins, M.J. Mendes, Light management with quantum nanostructured dots-in-host semiconductors, Light Sci. Appl. 10 (2021) 231. https://doi.org/10.1038/s41377-021-00671-x.

[2] M. Alexandre, 4-band k.p-based Method to Solve QD@Host Problems, (2022). https://github.com/perspe/qd-host (accessed November 3, 2022).

[3] The deal.II Finite Element Library, (n.d.). https://dealii.org/ (accessed November 3, 2022).

[4] S.I. Sadovnikov, A.I. Gusev, Structure and properties of PbS films, J. Alloys Compd. 573 (2013) 65–75. https://doi.org/10.1016/j.jallcom.2013.03.290.

[5] A. Luque, A.V. Mellor, Photon Absorption Models in Nanostructured Semiconductor Solar Cells and Devices, Springer International Publishing, 2015. https://doi.org/10.1007/978-3-319-14538-9.

[6] I. Ramiro, A. Martí, Intermediate band solar cells: Present and future, Prog. Photovolt. Res. Appl. 29 (2021) 705–713. https://doi.org/10.1002/pip.3351.

[7] E. Antolín, A. Martí, C.D. Farmer, P.G. Linares, E. Hernández, A.M. Sánchez, T. Ben, S.I. Molina, C.R. Stanley, A. Luque, Reducing carrier escape in the InAs/GaAs quantum dot intermediate band solar cell, J. Appl. Phys. 108 (2010) 064513. https://doi.org/10.1063/1.3468520.

[8] S. Brittman, E.C. Garnett, Measuring *n* and *k* at the Microscale in Single Crystals of $CH_3NH_3PbBr_3$ Perovskite, J. Phys. Chem. C 120 (2016) 616–620. https://doi.org/10.1021/acs.jpcc.5b11075.
